# Supplementary material for: BMP suppresses WNT to integrate patterning of orthogonal body axes in adult planarians
Source: bioRxiv. 2023 Jan 12:2023.01.10.523528. Preprint. [Version 1] doi: 10.1101/2023.01.10.523528 (PMC9882038; doi:10.1101/2023.01.10.523528)
Supplement: 1 — Fig. S1. wnt1 inhibits bmp4 expression in the posterior. FISH staining for bmp4 expression after 14 days of control or wnt1 RNAi. Inhibition of wnt1 results in elevated expression of bmp4 on the posterior midline of the animal (arrows). Bottom panels show enlargements. Scale bars are 150 μm. N = 5 animals. Fig. S2. Reduction of wnt1 expression domain through AP regenerative rescaling is not influenced by bmp4 inhibition. (A) FISH to detect wnt1 expression in regenerating tail fragments at 0, 18, and 96 hours after amputations conducted after 14 days of either control or bmp4 RNAi. Arrows indicate anterior-most wnt1+ cell detected along the dorsal midline for each timepoint and condition. Top panels show control animals undergoing early expansion of the dorsal midline wnt1 domain by 18 hours of regeneration followed by rescaling to reduce the domain to the tip of the animal by 96 hours. Bottom panel shows wnt1 expression dynamics in bmp4 RNAi, in which midline wnt1 expression was anterior expanded at the time of injury (0 hours), remained expanded at 18 hours of regeneration, and then restricted posteriorly by 96 hours, similar to control RNAi conditions. Therefore, BMP pathway modulation is unlikely to be responsible for the normal restriction of wnt1 by 96 hours in regenerating tail fragments. Scale bars represent 150 μm. (B) Graph showing the quantification of the length of the wnt1 domain relative to length of tail fragment. ****p<0.0001 by 2-tailed t-test and n.s. indicates p>0.05; N ≥ 3 animals. Box plots shows median values (middle bars) and first to third interquartile ranges (boxes); whiskers indicate 1.5× the interquartile ranges and dots are data points from individual animals. Fig. S3. bmp4 promotes midline identity and suppresses lateral identity. (A-C) FISH for dd23400, LaminB, and wnt5 following 28 days of control or bmp4 RNAi. Scale bars represent 300 μm. (A) Inhibition of bmp4 reduces dd23400 expression (arrows), particularly in the posterior. Right: [file NIHPP2023.01.10.523528v1-supplement-1.pdf]

bioRxiv preprint doi: https://doi.org/10.1101/2023.01.10.523528; this version posted January 12, 2023. The copyright holder for this preprint (which was not certified by peer review) is the author/funder, who has granted bioRxiv a license to display the preprint in perpetuity. It is made available under aCC-BY 4.0 International license.

Figure S1

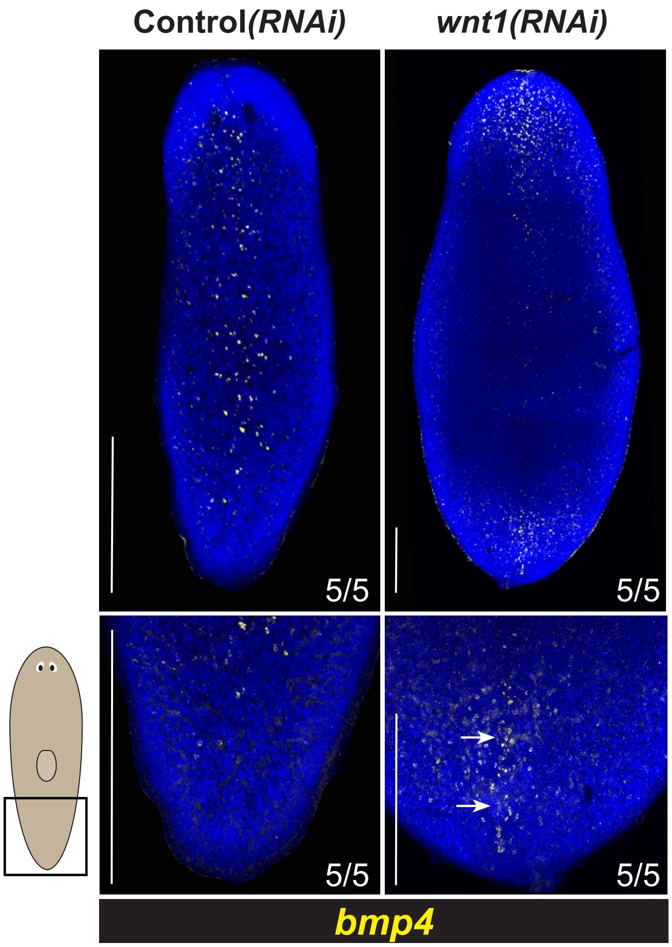

bioRxiv preprint doi: https://doi.org/10.1101/2023.01.10.523528; this version posted January 12, 2023. The copyright holder for this preprint (which was not certified by peer review) is the author/funder, who has granted bioRxiv a license to display the preprint in perpetuity. It is made available under aCC-BY 4.0 International license.

Figure S2

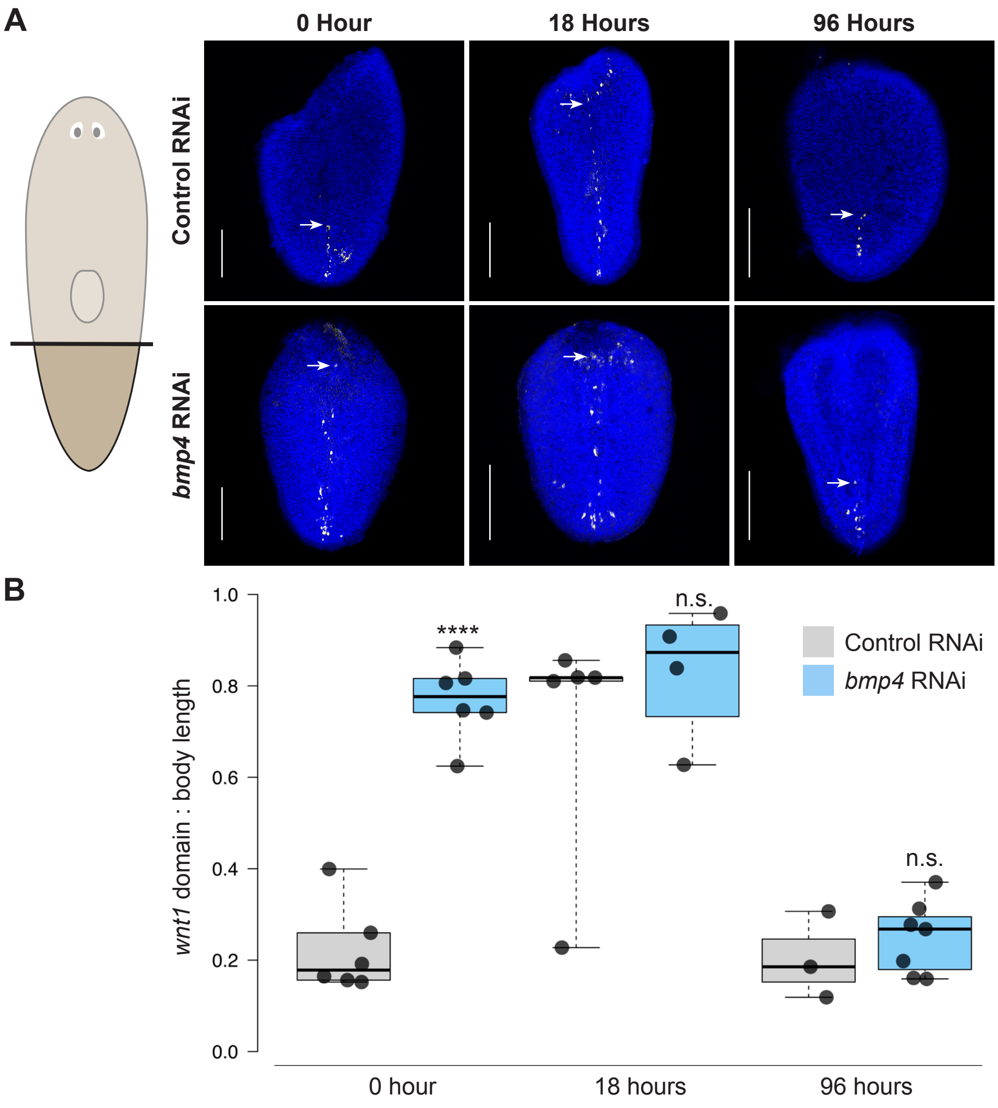

bioRxiv preprint doi: https://doi.org/10.1101/2023.01.10.523528; this version posted January 12, 2023. The copyright holder for this preprint (which was not certified by peer review) is the author/funder, who has granted bioRxiv a license to display the preprint in perpetuity. It is made available under aCC-BY 4.0 International license.

Figure S3

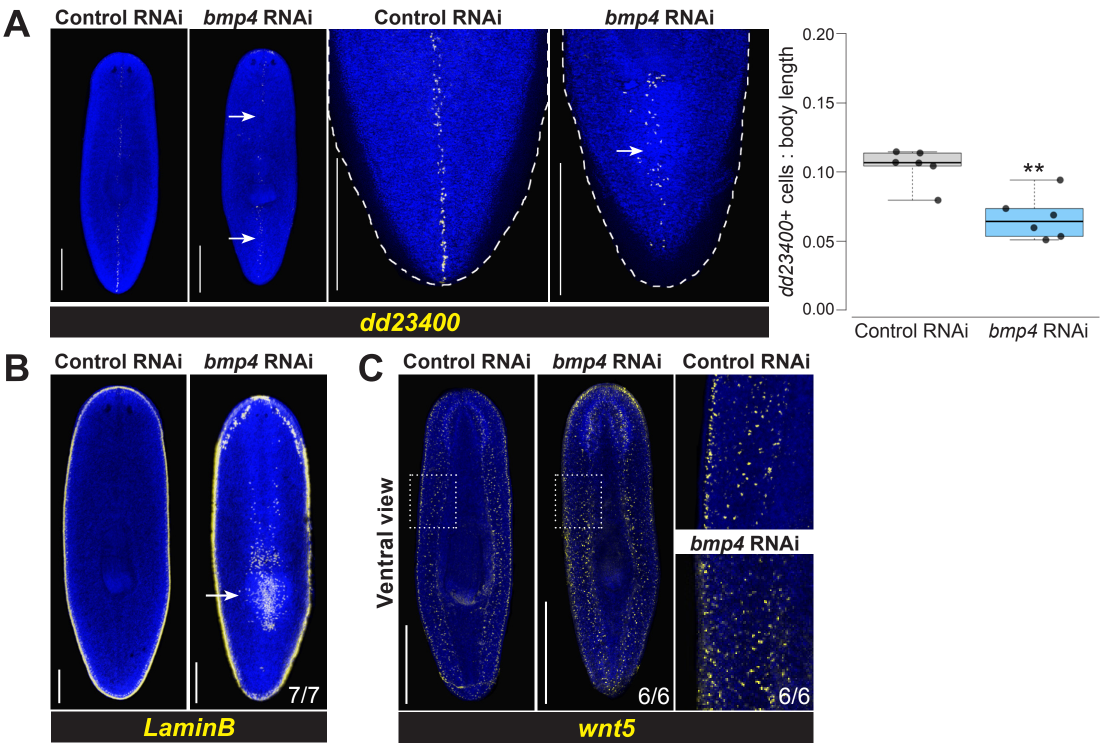

bioRxiv preprint doi: https://doi.org/10.1101/2023.01.10.523528; this version posted January 12, 2023. The copyright holder for this preprint (which was not certified by peer review) is the author/funder, who has granted bioRxiv a license to display the preprint in perpetuity. It is made available under aCC-BY 4.0 International license.

Figure S4

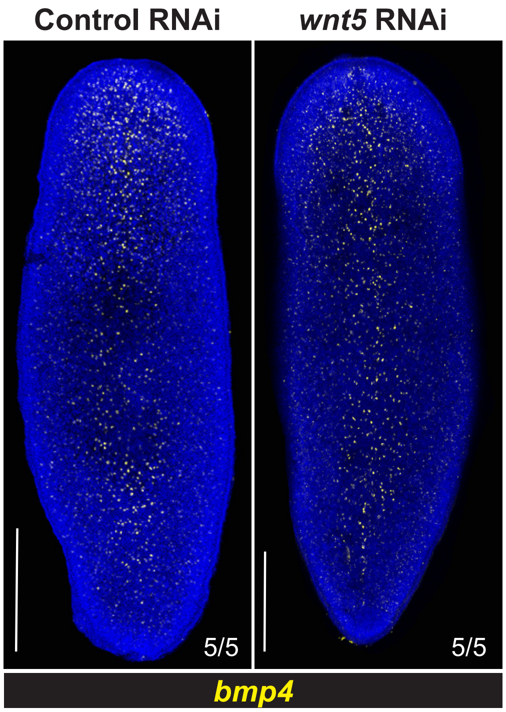

bioRxiv preprint doi: https://doi.org/10.1101/2023.01.10.523528; this version posted January 12, 2023. The copyright holder for this preprint (which was not certified by peer review) is the author/funder, who has granted bioRxiv a license to display the preprint in perpetuity. It is made available under aCC-BY 4.0 International license.

Table S1. Primer sequenes

Gene name	ddv6	Left Primer	Right Primer
bmp4	dd_Smed_v6_17402_0_1	TCCATCAGAGAAAGTTCGCAGT	ACGAATGTACAGTTTCAGTTGCA
bambi-2	dd_Smed_v6_23400_0_1	GCCCATCAGATGTCCGCTTA	TTTTCCAACACAGTGCAACACA
wnt1	dd_Smed_v6_28398_0_1	CCTCAAAATCGAATTTTACACTCA	TGGGACAAAAATAAAATTCCACA
smad1	dd_Smed_v6_6877_0_1	CATCTCCGGCTGTTAAACGG	CCACAAGAATAAAGCAAAATGGCA
smad4	dd_Smed_v6_1923_0_1	CTTCAAATTGCCGGGCGAAA	TCGAGTATCGCGCTGATTCC
tbx2/3	dd_Smed_v6_11693_0_1	GCTTCGTTTCTGCCGAGTTT	CGGATGGGTTTTGAATCGCG
nlg8	dd_Smed_v6_8738_0_1	CCTCCACGTGAATCCACAGT	TCGGTTTCCAGAACTGAGAAA
notum	dd_Smed_v6_24180_0_1	AAAATTTCTGAGGATCGAAAAA	TGAAGCTAGATTTATGTGAAAAACCA
sfrp-1	dd_Smed_v6_13985_0_1	TTGAATTCATGGAAATGACCAA	AATCAATGAAATGTTTTGTTGTGA
ndl-5	dd_Smed_v6_5102_0_1	ACAGTATTTCTTAACACGGGTCA	TGAACCATACGGAGCGGT
wntP-2	dd_Smed_v6_7326_0_1	TTAAATGTTCTAAGCCAAAACAACA	AAAACTTTTATGATCAATCTGAATGC
fzd4-1	dd_Smed_v6_11650_0_1	GGAATAGCCCAACTCACCAA	TGCCGAATTTAGTTGGAAGC
wnt11-1	dd_Smed_v6_14391_0_1	CATGAGCCAGTAAATGAAATGGT	TAGCACTGCGTTGGTGTTTG
wn11-2	dd_Smed_v6_16209_0_1	TTGATCGCATGAAAAATTACAAA	CCATTGCAATAAAATTGTCCA
kal-1	dd_Smed_v6_6746_0_1	CGAGTTCTGAACCTGGCTGT	GTTTCCCCTCCTGTGTTGAA
slit	dd_Smed_v6_12111_0_1	TCCGTGACAATCAGCTGCAA	AAGCCGATGATTGCCGAGAA
wnt5	dd_Smed_v6_15469_0_1	TCGGGGTGACCTTTTACTCA	TGAACCCATTGCAGTGAAAA
laminB	dd_Smed_v6_3065_0_1	AGTTCAACTCCTGGCGCTAC	CTGAACATTCCGGATCACCT
